# Optical Control of CD8^+^ T Cell Metabolism and Effector Functions

**DOI:** 10.3389/fimmu.2021.666231

**Published:** 2021-06-03

**Authors:** Andrea M. Amitrano, Brandon J. Berry, Kihong Lim, Kyun-Do Kim, Richard E. Waugh, Andrew P. Wojtovich, Minsoo Kim

**Affiliations:** ^1^ Department of Pathology, University of Rochester Medical Center, Rochester, NY, United States; ^2^ Department of Microbiology and Immunology, David H. Smith Center for Vaccine Biology and Immunology, University of Rochester Medical Center, Rochester, NY, United States; ^3^ Department of Pharmacology and Physiology, University of Rochester Medical Center, Rochester, NY, United States; ^4^ Center for Convergent Research of Emerging Virus Infection, Korea Research Institute of Chemical Technology, Daejeon, South Korea; ^5^ Department of Biomedical Engineering, University of Rochester Medical Center, Rochester, NY, United States; ^6^ Department of Anesthesiology and Perioperative Medicine, University of Rochester Medical Center, Rochester, NY, United States

**Keywords:** optogenetics, metabolism, T cell migration, effector T cell, cancer immunotherapy

## Abstract

Although cancer immunotherapy is effective against hematological malignancies, it is less effective against solid tumors due in part to significant metabolic challenges present in the tumor microenvironment (TME), where infiltrated CD8^+^ T cells face fierce competition with cancer cells for limited nutrients. Strong metabolic suppression in the TME is often associated with impaired T cell recruitment to the tumor site and hyporesponsive effector function *via* T cell exhaustion. Increasing evidence suggests that mitochondria play a key role in CD8^+^ T cell activation, effector function, and persistence in tumors. In this study, we showed that there was an increase in overall mitochondrial function, including mitochondrial mass and membrane potential, during both mouse and human CD8^+^ T cell activation. CD8^+^ T cell mitochondrial membrane potential was closely correlated with granzyme B and IFN-γ production, demonstrating the significance of mitochondria in effector T cell function. Additionally, activated CD8^+^ T cells that migrate on ICAM-1 and CXCL12 consumed significantly more oxygen than stationary CD8^+^ T cells. Inhibition of mitochondrial respiration decreased the velocity of CD8^+^ T cell migration, indicating the importance of mitochondrial metabolism in CD8^+^ T cell migration. Remote optical stimulation of CD8^+^ T cells that express our newly developed “OptoMito-On” successfully enhanced mitochondrial ATP production and improved overall CD8^+^ T cell migration and effector function. Our study provides new insight into the effect of the mitochondrial membrane potential on CD8^+^ T cell effector function and demonstrates the development of a novel optogenetic technique to remotely control T cell metabolism and effector function at the target tumor site with outstanding specificity and temporospatial resolution.

## Introduction

Despite recent advances in the treatment of malignant tumors, cancer continues to be a widespread disease and a leading cause of death. CD8^+^ T cell-based immunotherapy has emerged as a potential treatment for several types of cancer (1). For example, chimeric antigen receptor-transduced T cells (CAR-T cells) have been designed to augment CD8^+^ T cell antitumor activity and are extremely successful in treating hematological malignancies (2, 3). However, this approach is ineffective against solid tumors due in part to the immunosuppressive tumor microenvironment (TME) which promotes T cell exhaustion (4–6).

The TME presents many metabolic challenges to CD8^+^ T cell energy production by depleting oxygen, glucose, and other metabolites as well as limiting the uptake of key nutrients *via* the expression of inhibitory ligands (6–8). Tumor-infiltrating CD8^+^ T cells (TILs) often show decreased mitochondrial mass and function and, thus, suppressed mitochondrial ATP production (5). Previous studies have shown that CD8^+^ T cells are metabolically flexible and undergo metabolic reprogramming throughout each stage of T cell activation, which includes altering dependence on mitochondria for energy production (9–15). Here, we show an increase in both mitochondrial mass and membrane potential during CD8^+^ T cell activation. In addition, CD8^+^ T cells exhibit increased oxygen consumption and glycolysis during migration on intercellular adhesion molecule 1 (ICAM-1), suggesting that migrating CD8^+^ T cells require elevated metabolism to fulfill the energy requirements of cell motility. Reprogramming effector CD8^+^ T cell metabolism to increase mitochondrial mass and function has been shown to improve antitumor activity (5). In this study, we further characterized a novel tool, a photoactivatable proton pump called “OptoMito-On,” to increase mitochondrial ATP production in CD8^+^ T cells with the aim of improving T cell immunotherapies for solid malignancies.

## Results

### Mitochondrial Function Increases During *In Vitro* CD8^+^ T Cell Activation

**Figure 1:**
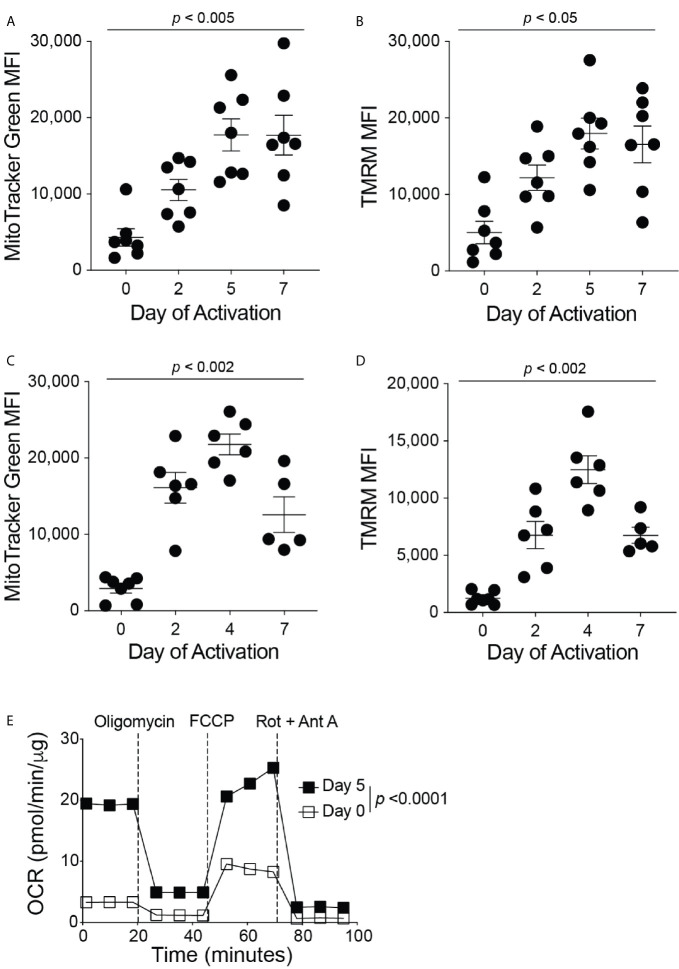
Mitochondrial function increases during CD8^+^ T cell activation. Flow cytometry analysis of **(A)** MitoTracker Green FM, reflecting mitochondrial mass, and **(B)** Tetramethylrhodamine (TMRM), reflecting mitochondrial membrane potential, displayed as mean fluorescence intensity (MFI) during human CD8^+^ T cell activation (n = 7 donors). Flow cytometry analysis of **(C)** MitoTracker Green FM and **(D)** TMRM shown in MFI during mouse CD8^+^ T cell activation (n = 5-7 mice). **(E)** The oxygen consumption rate (OCR), measured with the Seahorse MitoStress Test, of naïve (Day 0, PLL + CCL21 coated wells) and activated (Day 5, PLL + CXCL12 coated wells) CD8^+^ T cells, normalized to protein content with a BCA assay; data shown as mean ± SEM (Representative of two independent experiments, n = 10 wells per group, error bars fall within symbols). All data shown as mean ± SEM. **(A, B)** Repeated measures one-way ANOVA with Bonferroni’s post-test. **(C, D)** Ordinary one-way ANOVA with Bonferroni’s post-test. **(E)** 2way ANOVA with Bonferroni’s post-test.

The current therapeutic T cell manufacturing process (both for TILs and CARs) includes the activation and expansion of T cells from patient apheresis products using CD3/CD28 beads at a clinical scale with high concentrations of IL-2 (16). To investigate the mitochondrial dynamics throughout the *in vitro* T cell manufacturing process, we isolated all T cell types from the blood of healthy human donors and activated these T cells *in vitro* with anti-CD3 and anti-CD28 antibodies in the presence of IL-2. On days 0, 2, 5, and 7 of activation, the mitochondrial mass and membrane potential of the T cells was measured by flow cytometry analysis of MitoTracker Green FM and Tetramethylrhodamine (TMRM) signals, respectively (**Figures 1A, B**). During the activation of T cells, there was an increase in mitochondrial mass and mitochondrial membrane potential from Day 0 through Day 5, and both mitochondrial signals plateaued after Day 5 (**Figures 1A, B**). These results suggest the importance of mitochondrial function in order to support T cell activation. To determine if a similar trend in mitochondrial function is seen during the activation of mouse T cells, we isolated primary CD8^+^ T cells from the spleen and lymph nodes of C57BL/6J mice. Again, the isolated CD8^+^ T cells were activated with anti-CD3 and anti-CD28 antibodies in the presence of IL-2. On days 0, 2, 4, and 7 of activation, CD8^+^ T cells were stained with MitoTracker Green FM or TMRM and analyzed by flow cytometry (**Figures 1C, D**). Similar to what was seen in human T cells, mouse CD8^+^ T cells exhibit an increase in mitochondrial mass and mitochondrial membrane potential from Day 0 through Day 4, though a decrease or plateau was observed on Day 7 (**Figures 1C, D**), suggesting that increases in mitochondrial mass and function coincide with CD8^+^ T cell proliferation and activation. In addition, activated mouse CD8^+^ T cells exhibited a higher basal oxygen consumption rate (OCR) than naïve CD8^+^ T cells, further demonstrating the requirement for increased mitochondrial function during CD8^+^ T cell activation (**Figure 1E**). Glycolysis, as measured by the extracellular acidification rate (ECAR) was also elevated in activated CD8^+^ T cells, supporting previous studies that showed the upregulation of aerobic glycolysis immediately after CD8^+^ T cell activation with anti-CD3 and anti-CD28 **(Supplementary Figure 1A**) (14, 17, 18). In addition, activated CD8^+^ T cells had a higher maximum OCR, without changes in the spare respiratory capacity of naïve and activated CD8^+^ T cells (**Supplementary Figures 1B–D**). To ensure that the concentrations of the metabolic inhibitors used in the test are not harmful to T cells in these conditions, we performed flow cytometry on activated mouse CD8^+^ T cells treated with the inhibitors and stained for Annexin-V. There was no increase in the percentage of T cell death following treatment with the inhibitors (**Supplementary Figure 1E**).

### Mitochondrial Respiration Is Important for Cytokine Production and Migration of CD8^+^ T Cells

**Figure 2:**
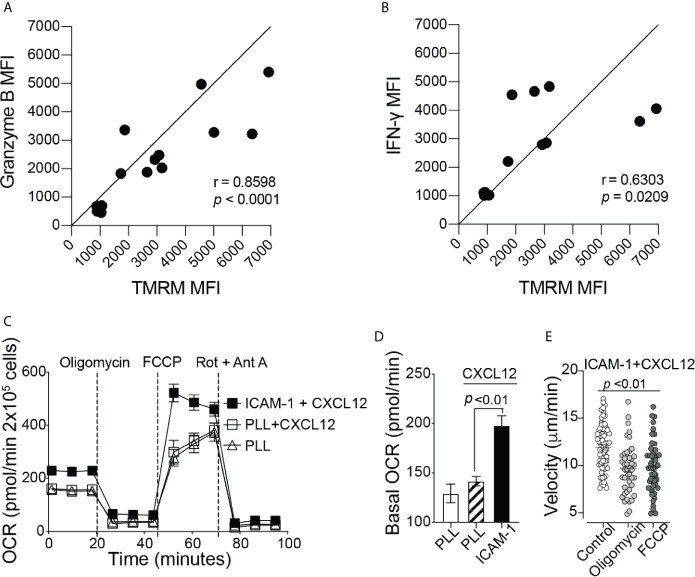
Mitochondrial respiration is important for cytokine production and migration of CD8^+^ T cells. Flow cytometry MFI of **(A)** Granzyme B and **(B)** IFN-γ production throughout mouse CD8^+^ T cell activation, shown as a function of TMRM MFI (n = 6 mice). **(C)** The complete OCR trace and **(D)** the basal OCR measured with the Seahorse MitoStress Test, of activated CD8^+^ T cells on Poly-L-lysine ± CXCL12, or migrating on ICAM-1 with CXCL12 (Representative of three independent experiments). **(E)** The velocity of activated CD8^+^ T cells migrating on ICAM-1 + CXCL12. Treated cells (2 µM oligomycin or 2 µM FCCP) were incubated with the drug for 20 minutes before the start of the 20-minute movie (Representative of three experiments). Migration data analyzed with Volocity software. **(A, B)** Pearson’s correlation. **(D, E)** Data shown as mean ± SEM and analyzed by one-way ANOVA with a Bonferroni post-test.

To elicit an immune response against cancer, CD8^+^ T cells need to release effector molecules, such as Granzyme B and IFN-γ. We found a positive correlation between TMRM signal with granzyme B and IFN-γ expression (**Figures 2A, B**), indicating that the increase in mitochondrial membrane potential may be important for supporting CD8^+^ T cell effector functions. Efficient trafficking of T cells to the target tumor site is key in order to perform anticancer effector functions (19, 20). Therefore, in addition to effector functions, additional energy is likely required for CD8^+^ T cell migration. To test this hypothesis, we next investigated whether mitochondrial metabolism is important for CD8^+^ T cell migration. Activated CD8^+^ T cells were plated on wells coated with poly-L-lysine (PLL), with or without the addition of CXCL12, to confine the CD8^+^ T cells in place or on ICAM-1 + CXCL12 coated wells to facilitate cell migration mediated by lymphocyte function-associated antigen-1 (LFA-1) and ICAM-1 interactions (21). CD8^+^ T cells on ICAM-1 + CXCL12-coated surfaces showed a significant increase in the basal and maximum OCR, as well as the basal ECAR, without changes in the spare respiratory capacity, compared to those on PLL-coated surfaces (**Figures 2C, D** and **Supplementary Figures 2A–D**). Our data suggest that CD8^+^ T cells migrating on ICAM-1 increase ATP production through both mitochondrial respiration and glycolysis to supply the additional energy required for cell migration. In addition, when CD8^+^ T cells were treated with mitochondrial respiration inhibitors, such as oligomycin, an ATP synthase inhibitor, or FCCP, a protonophore that uncouples OxPhos, there was a significant decrease in the velocity of CD8^+^ T cells migrating on ICAM-1 + CXCL12, further demonstrating the importance of mitochondrial function in active CD8^+^ T cell migration (**Figure 2E**).

CD8^+^ T cells have increased levels of mitochondrial respiration to support the energetic demands of cell migration (**Figure 2**); thus, a hypoxic TME may suppress CD8^+^ T cell migration at the tumor site by preventing sufficient ATP production to fuel migration. To test this hypothesis under TME-like hypoxic conditions, we used cobalt (II) chloride hexahydrate (CoCl_2_) (22). Treatment of CD8^+^ T cells with CoCl_2_ reduced the basal and maximum OCR in a dose-dependent manner (**Supplementary Figures 3A, B**). In addition, CoCl_2_ caused a reduction in the overall track velocity and the percentage of total migrating CD8^+^ T cells on ICAM-1 + CXCL12-coated wells (**Supplementary Figures 3E–G**). It should be noted that CoCl_2_ had no impact on CD8^+^ T cell survival during the experiment, and there was no change in Annexin-V-positive cells treated with CoCl_2_ (**Supplementary Figure 3H**). Although the effect was minimum, CoCl_2_ also caused a decrease in the ECAR (**Supplementary Figures 3C, D**). Therefore, we cannot completely exclude the possibility that some of the outcomes of the cell migration assay may have resulted from the reduction in glycolysis with CoCl_2_ treatment.

### Optogenetic Regulation of Mitochondrial Membrane Potential

**Figure 3:**
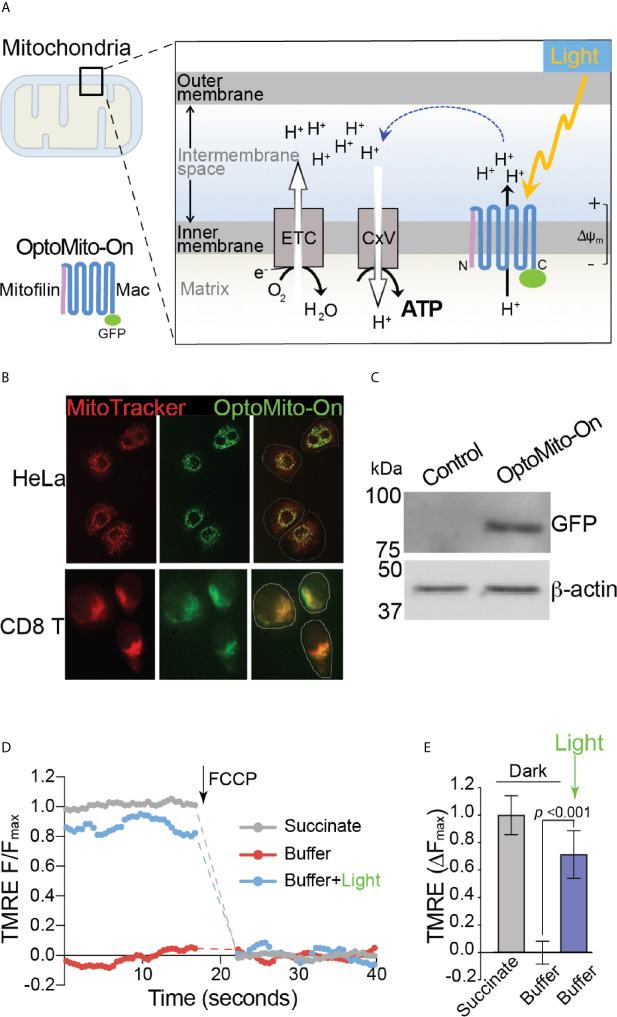
Characterization of OptoMito-On. **(A)** Illustration of photoactivatable oxidative phosphorylation by OptoMito-On construct. **(B)** Images of HeLa cells and CD8^+^ T cells expressing OptoMito-On and stained with MitoTracker Red CMXRos. **(C)** Immunoblot comparing control HEK293T cell lysate and HEK293T OptoMito-On cell lysate. The full-length OptoMito-On construct is 82 kDa and probed for with an anti-GFP antibody. β-actin is used as loading control. Both images are from the same lanes on one membrane. **(D)** Representative TMRE fluorescence trace of isolated mitochondria from OptoMito-On expressing HEK293T cells before and after addition of FCCP. Dashed lines indicate where FCCP was added. **(E)** Quantification of change in TMRE fluorescence. Data shown as mean ± SEM and analyzed by One-Way ANOVA with a Bonferroni post-test (n = 4).

Although delivery of an immune-enhancing or a metabolism-promoting molecule to overcome local immunosuppression has been proposed, the full potential of this approach is limited by aberrant activation of the host immune system and nonspecific stimulation of tumor growth and metastasis. To overcome the metabolic challenges in the TME and to selectively enhance CD8^+^ T cell functions, we developed a genetically encoded photoactivatable proton pump (“OptoMito-On”) that is expressed in the inner mitochondrial membrane (**Figure 3A**) (23). The OptoMito-On construct consists of the light-driven proton pump, ‘Mac,’ that was derived from the fungus *Leptosphaeria maculans* (24) and is fused to the N-terminal mitochondrial targeting domain of the mitochondrial inner membrane protein Mitofilin (25), which delivers and orients Mac in the membrane (**Figure 3A**) (26, 27). During OxPhos, electrons from NADH and FADH_2_, products of cellular metabolism, enter the mitochondrial electron transport chain (ETC), located in the inner mitochondrial membrane. As electrons are transferred through the ETC to oxygen, protons are pumped from the mitochondrial matrix to the intermembrane space, thereby generating a protonmotive force that is composed of an electrical (Δψ_m_) and chemical (ΔpH) gradient across the inner membrane (**Figure 3A**) (28). Importantly, the establishment of this gradient requires both oxygen and reducing equivalents generated by cellular metabolism. ATP synthase (complex V) utilizes the protonmotive force to phosphorylate ADP to ATP. We predicted that OptoMito-On would mimic the function of the ETC by pumping protons to the intermembrane space upon stimulation with light and thus generate ATP *via* ATP synthase in the absence of sufficient oxygen and substrates (common features in the TME).

We first transfected HeLa and mouse CD8^+^ T cells with the OptoMito-On construct and labeled cells with MitoTracker to specifically stain the mitochondria. Confocal microscopy confirmed that there was a high degree of overlap between GFP signals (OptoMito-On) and red fluorescence signals (mitochondria), indicating that OptoMito-On was successfully expressed in the mitochondria (**Figure 3B** and **Supplementary Figure 4**). Expression of the full-length OptoMito-On construct was further confirmed by Western blot analysis of cell lysates prepared from HEK293T cells that expressed OptoMito-On (82 kDa) (**Figure 3C**).

Based on our model (**Figure 3A**), stimulation of OptoMito-On with light results in polarization of the mitochondrial inner membrane. To determine whether OptoMito-On reaches the inner mitochondrial membrane in the correct orientation and to confirm the functional responsiveness of OptoMito-On, we measured the mitochondrial membrane potential (Δψ_m_) using TMRE (29, 30). We first isolated mitochondria from HEK293T cells expressing OptoMito-On and treated them with succinate, a complex II ETC substrate, to generate a maximal mitochondrial membrane potential as a positive control (**Figure 3D**). In the absence of succinate (a negative control), the mitochondria did not generate a membrane potential. However, stimulation with 540-600 nm light was sufficient to rescue the mitochondrial membrane potential in the absence of succinate, indicating the expression and correct orientation of functional OptoMito-On in mitochondria (**Figures 3D, E**).

**Figure 4:**
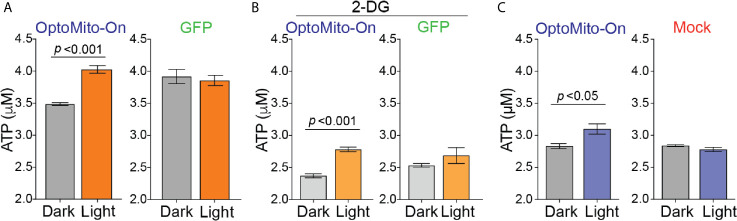
Activation of OptoMito-On increases ATP production. **(A)** HEK293T cells expressing OptoMito-On or GFP were illuminated with 590 nm light for 2 hours, followed by a luciferase-based ATP assay. **(B)** Same set-up as in **(A)**, except HEK293T cells were treated with 10 mM 2-DG for 2 hours prior to illumination. **(C)** Activated CD8^+^ T cells were sorted based on GFP expression. GFP negative cells were used as the mock control. CD8^+^ T cells received 590 nm light for 30 minutes, followed by a luciferase-based ATP assay. All data shown as mean ± SEM and analyzed by an unpaired t-test (n = 4-7).

To test whether mitochondrial polarization driven by light-activated proton transport leads to enhanced ATP production (**Figure 3A**), we transfected HEK293T cells and mouse CD8^+^ T cells with OptoMito-On, illuminated the cells (590 ± 10 nm, 0.32 mW/mm2 at 1 Hz), and immediately lysed them to detect ATP. 2-Deoxy-D-glucose (2-DG), a glucose analog, was used to inhibit glycolysis and thus mimic the glucose-deficient TME. Optical stimulation of OptoMito-On expressing HEK293T cells with or without 2-DG treatment yielded a significant increase in ATP compared with that of cells cultured in the dark (**Figures 4A, B**). Stimulation of GFP-expressing cells with light did not alter ATP levels, indicating that stimulation with light at 590 nm was not detrimental to cell viability. We observed a similar outcome in OptoMito-On expressing CD8^+^ T cells (**Figure 4C**). However, unlike HEK293T cells, light stimulation of 2-DG treated OptoMito-On expressing T cells failed to increase ATP production (**Supplementary Figure 5A**). In earlier studies, 2-DG treatment of effector T cells showed a decrease in mitochondrial respiration as well as decreases in other effector functions, including cytokine production and cytolytic activity (13, 31). Therefore, we speculate that 2-DG treatment of OptoMito-On expressing CD8^+^ T cells may have broader effects on overall metabolic functions than in HEK293T cells, preventing the light-induced increase in mitochondrial ATP production. In combination with the isolated mitochondria data, these results demonstrate that OptoMito-On can be expressed in cells and facilitates photoactivatable control of the mitochondrial membrane potential.

CD8^+^ T cells are metabolically flexible and are able to adjust their reliance on metabolic pathways depending on the nutrient availability in their immediate environment (17). To determine whether boosting mitochondrial ATP production with OptoMito-On in activated CD8^+^ T cells mediates changes in T cell glycolysis, we measured glucose consumption. Illumination of OT-I T cells with 530 nm light caused no change in the glucose consumption rate of both GFP and OptoMito-On expressing T cells (**Supplementary Figure 5B**). These results suggest that increasing mitochondrial ATP production with OptoMito-On does not impact the rate of glycolysis, at least during a one-hour light activation period.

### OptoMito-On Enhances CD8^+^ T Cell Functions

**Figure 5:**
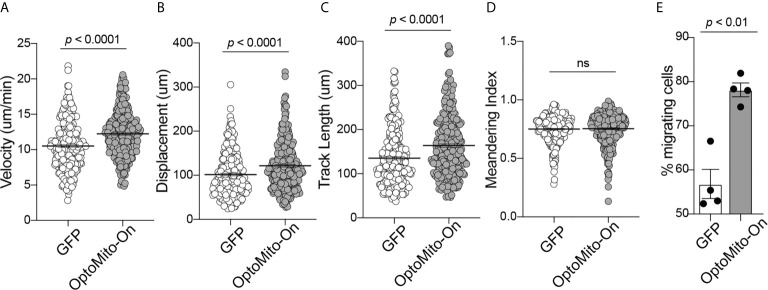
Activation of OptoMito-On increases CD8^+^ T cell migration. The velocity **(A)**, displacement **(B)**, track length **(C)**, meandering index **(D)**, and percent migrating cells **(E)** of Mock (or GFP) and OptoMito-On expressing Day 4 CD8^+^ T cells migrating on ICAM-1 + CXCL12. The percentage of migrating cells was calculated as the number of cells migrating 5-20 µm/min divided by the total number of cells in the field of view during the 20-minute movie. All movies received 500 nm illumination and were analyzed with Volocity software. All data shown as mean ± SEM and analyzed by a two-tailed unpaired t-test with Welch’s correction (**A–D**: includes four independent experiments, Mock: 271 cells, OptoMito-On: 312 cells; **E** : n = 4 movies on the same day). ns, not significant.

The roles of the mitochondrial membrane potential and cell metabolism in the regulation of T cell functions are not completely understood. To investigate the impact of mitochondrial ATP production induced by stimulation of OptoMito-On in CD8^+^ T cell migration, CD8^+^ T cells were transduced with either OptoMito-On or control GFP retrovirus, then allowed to migrate on an ICAM-1 + CXCL12 coated well in the presence (light) or absence (dark) of light stimulation (**Movies 1** and **2**). CD8^+^ T cells expressing OptoMito-On showed an increase in cell velocity, cell displacement, and track length compared to mock cells expressing GFP or that were negative for OptoMito-On, with no change in the meandering index (**Figures 5A–D**). Additionally, there was a large increase in the overall percentage of migrating CD8^+^ T cells expressing OptoMito-On compared to those expressing GFP (**Figure 5E**). These results indicate that although stimulation of OptoMito-On with light may not improve the ability of T cells to become polarized towards chemoattractants, but increasing mitochondrial ATP production with OptoMito-On is able to enhance overall CD8^+^ T cell migration, which we have shown requires increased mitochondrial metabolism.

In CD4^+^ T cells, it has been shown that upon chemokine stimulation, an increase in cytosolic calcium stimulates mitochondrial ATP production and subsequent ATP release to activate autocrine signaling through P2X4 receptors to support cell migration (32). Additionally, mitochondria can localize to the uropod during T cell migration to supply ATP for cytoskeletal motor proteins (33). To further identify key cytoplasmic molecules that regulate OptoMito-On-mediated CD8^+^ T cell migration, we performed an antibody array that specifically detects various cytoskeletal proteins.

**Figure 6:**
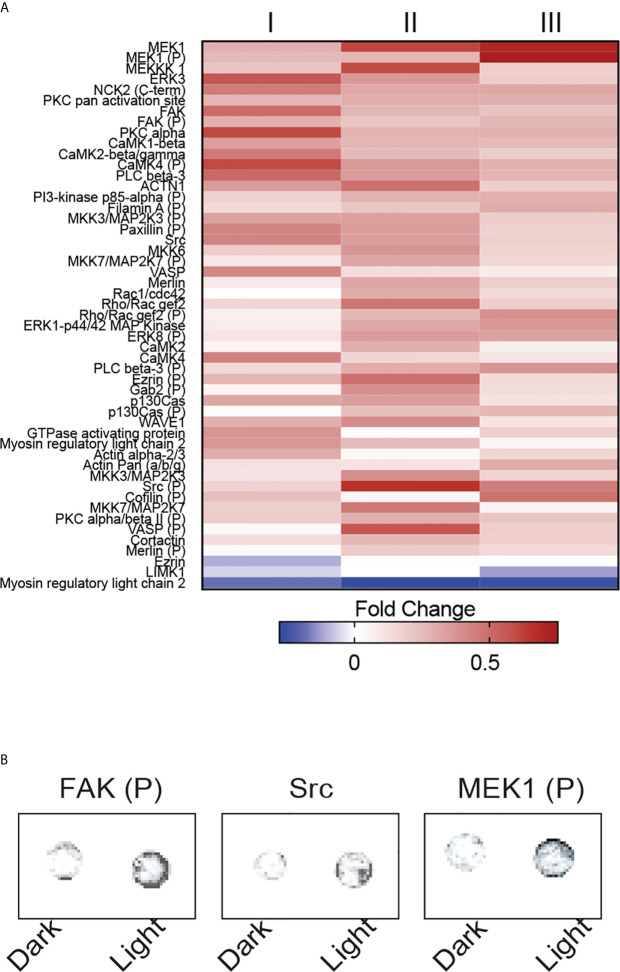
OptoMito-On activation increases cytoskeletal rearrangements. **(A)** Heat map showing the fold change in protein expression from the dark to light conditions of the Full Moon BioSystems Cytoskeleton Phospho antibody array (n = 3). **(B)** Representative antibody array images of a few proteins of interest, FAK, Src, and MEK1.

Among the 141 antibody targets that were screened, the expression of 51 molecules were considerably increased or decreased after the activation of OptoMito-On by light in migrating CD8^+^ T cells (**Figure 6A**). Cell migration is a complex process that involves the coordination of many signaling pathways. For example, dynamic regulation of the phosphorylation of focal adhesion kinase (FAK) and MEK1, as well as the expression of Src are involved in regulation of cell adhesion turnover at both the leading edge and in the rear of migrating cells (34).

Activation of FAK and Src promotes formation of a signaling complex that leads to further downstream signaling, such as the MAP kinase pathway which includes MEK1 (35). Consistently, the phosphorylation of FAK and MEK1 and the protein expression of Src were increased after stimulation of CD8^+^ T cells that express OptoMito-On with light compared to cells in the dark (**Figure 6B**). Therefore, our data indicates that the increase in mitochondrial ATP production by light activation of OptoMito-On is sufficient to drive heightened levels of dynamic cytoskeleton rearrangements, such as increased cell adhesion turnover, leading to improved CD8^+^ T cell migration. Importantly, there is still debate about whether activated CD8^+^ T cells utilize focal adhesion independent or dependent motility (36, 37). Integrin activation is tightly regulated by interaction of cytoplasmic domains with signaling proteins. FAK has been shown to be a key inside-out signaling molecule that mediates LFA-1 ligation and TCR engagement in T cells (38–41)

**Figure 7:**
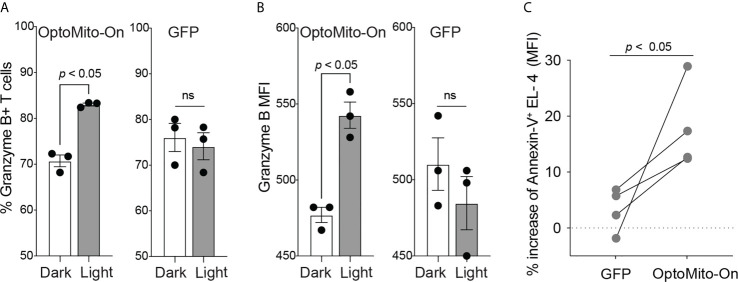
OptoMito-On activation increases CD8^+^ T cell effector functions. Flow cytometry results from an 18-hour light activation of GFP or OptoMito-On expressing CD8^+^ T cells looking at the percentage of Granzyme B+ cells **(A)** and Granzyme B MFI **(B)** (n = 3). **(C)** Flow cytometry results of a 3-hour co-culture of OVA pulsed EL-4 cells with GFP or OptoMito-On expressing OT-I T cells. The graph shows the percent change of Annexin-V MFI of EL-4 cells from the dark to light condition. OT-I T cells were either kept in the dark or received 16-17 hours of 530 nm light before the addition of EL-4 cells. All data shown as mean ± SEM, A-B analyzed by One-Way ANOVA with a Bonferroni post-test, C analyzed by one-tailed paired t test (**A, B**: representative of two experiments, **C**: four independent experiments). ns, not significant.

In addition to cell migration, overnight illumination of OT-I CD8^+^ T cells, isolated from OT-I T cell receptor transgenic mice, that express OptoMito-On significantly increased the percentage of granzyme B positive cells and the average mean fluorescence intensity of intracellular granzyme B (**Figures 7A, B**). These results indicate the feasibility of remote activation of T cell effector function by light stimulation of mitochondrial ATP production. Interestingly, light activation of OptoMito-On expressing T cells failed to increase IFN-γ production. It may be possible that IFN-γ production during T cell stimulation overrides light-induced cytokine production. In addition, while granzyme B and IFN-γ can be expressed simultaneously by T cells, their kinetics and relative levels of expression can vary during activation. Furthermore, the genes encoding granzyme B and IFN-γ may be differentially regulated in activated CD8^+^ T cells by mitochondria OxPhos.

In order to further investigate the ability of OptoMito-On to improve CD8^+^ T cell effector function, we performed a co-culture killing assay with OptoMito-On or GFP expressing OT-I T cells and OVA loaded murine lymphoma EL-4 cells. Light stimulation of OptoMito-On expressing OT-I T cells caused a significant increase in the expression of Annexin-V in EL-4 cells compared to GFP expressing T cells (**Figure 7C**). Our data suggest that illuminated OptoMito-On OT-I T cells are able to induce robust apoptosis of EL-4 cells, which correlates with the increased granzyme B production after light stimulation (**Figures 7A, B**). Therefore, OptoMito-On is capable of selectively improving the cytotoxic functions of effector CD8^+^ T cells without augmenting the metabolism of target cells or other immunosuppressive cells at the tumor site.

## Discussion

Cellular metabolism involves a series of reactions that ultimately breakdown nutrients into usable energy. Under normal conditions, quiescent, resting T cells primarily utilize OxPhos to generate the energy source ATP. Cancer cells, however, reprogram their metabolic patterns from OxPhos to relying largely on aerobic glycolysis in order to support cell proliferation. This aerobic form of glycolysis, known as the “Warburg effect,” is useful for the rapid generation of ATP, the production of metabolic intermediates that are required for cell proliferation, and also for the survival of malignant cells in the hypoxic TME (42, 43). Similarly, while naïve T cells are relatively quiescent and rely primarily on OxPhos, CD8^+^ T cells undergo metabolic reprogramming during activation by shifting their metabolic pathways to utilize both glycolysis and OxPhos to meet the demands of clonal expansion and effector functions following antigen stimulation (11, 15, 44). The fact that both cancer cells and CD8^+^ T cells utilize overlapping forms of metabolism in the TME is an important obstacle to overcome for the development of effective immunotherapies because cancer cells will likely outcompete CD8^+^ T cells for any available nutrients (6). We have shown that mitochondrial function is required for CD8^+^ T cell activation, cytokine production, and migration, further supporting our rationale of targeting mitochondrial metabolism as a way to improve adoptive T cell therapy outcomes. Our study characterizes the use of a novel optogenetic tool, OptoMito-On, in mouse CD8^+^ T cells to remotely control T cell mitochondrial metabolism, migration, and effector function with outstanding specificity and temporospatial resolution.

Metabolic reprogramming of cells is commonly studied through the global administration of drugs that lack target selectivity, and it has not been possible to specifically regulate metabolic pathways only in a selected cell type *in vivo*, in a reversible manner, and at a precise location and time. For the same reason, although there is increasing evidence that metabolism can affect the survival and antitumor function of T cells, the development of a cell-specific and clinically feasible method to generate T cells with favorable metabolic features has proven challenging. Here, we utilized OptoMito-On to circumvent this limitation by directly and selectively modulating T cell OxPhos and studied the effects of mitochondrial membrane potential on effector CD8^+^ T cell function. Our results indicate that enhancing the overall mitochondrial membrane potential improves T cell functions by increasing CD8^+^ T cell migration and effector molecule production. This new optogenetic approach highlights the development and application of optogenetic techniques to control T cell functions using light stimulation, thus providing a unique opportunity to understand the fundamental mechanisms of T cell metabolic function in many diseases.

Recent studies demonstrate important functions of OxPhos for cell migration (45–47). In naïve T cells, chemokine stimulation under a confined 3D condition induces actin retrograde flow and cellular elongation, suggesting the presence of force-generating cytoskeletal processes regulated by chemokine signaling (48). Our results on an ICAM-1 coated 2D surface showed that basal OCR of activated CD8^+^ T cells is significantly increased compared to T cells on a PLL coated surface. During T cell migration on ICAM-1 substrate, force is exerted on the cytoplasmic domain of LFA-1, while the extracellular domain binds to ICAM-1 (49). These forces exerted on integrins are critical for LFA-1 mediated adhesion at the leading edge of migrating cells (50). Therefore, LFA-1 binding with ICAM-1 may prompt an increase in mitochondrial respiration to support the increased energy demands of migrating T cells. Activated CD8^+^ T cells on PLL did not have an increase in OCR in the presence of CXCL12 treatment. PLL may prevent CD8^+^ T cell polarization and elongation, thus impairing F-actin polymerization (33).

CD8^+^ T cell metabolic pathways are tightly regulated to fuel their optimum effector function, but tumor-infiltrating T cells often show an overall phenotype of metabolic insufficiency in the strong immunosuppressive TME due to a persistent loss of mitochondrial function (5, 7, 51). Therefore, a better understanding of CD8^+^ T cell mitochondrial function holds promise for the development of novel approaches to enhance T cell effector function, promote memory formation, and thus improve immunotherapy outcomes. This study further illustrates the importance of mitochondrial metabolism in CD8^+^ T cell effector functions and identifies the mitochondrial membrane potential as an ideal target to improve T cell therapy outcomes. T cells in the hypoxic TME have a decreased ATP/AMP ratio and display mitochondrial dysfunction, which correlates with a reduction in cytokine production. The treatment of chronically stimulated T cells with antioxidants to reduce mitochondrial oxidative stress was shown to reverse mitochondrial dysfunction and rescue effector T cell function, including IFN-γ, TNF, and Granzyme B production (52, 53). Therefore, the use of OptoMito-On to boost the mitochondrial membrane potential in chronically stimulated T cells can be beneficial in the TME to increase cytokine production in tumor-infiltrated T cells. We further predict that with different light intensities, gradual titration of metabolic activation to an appropriate therapeutic level may be possible, resulting in superior memory formation without T cell exhaustion or cell death due to overactivation of mitochondrial activity. The metabolic plasticity exhibited by CD8^+^ T cells (OxPhos *vs.* glycolysis) is likely essential for their function. Therefore, it is also possible that long-term stimulation with OptoMito-On may eventually suppress CD8^+^ T cell activity *in vivo* by circumventing this plasticity. We predict that the transition is required only for activation from quiescence and not for the ultimate effector functions of CD8^+^ T cells. Adverse effects (e.g., increased cell death) from stimulation of the mitochondrial membrane potential for a long period of time *in vivo* could lead to the development of a potential control mechanism that can remove excess adoptively transferred CD8^+^ T cells and ultimately decrease potential side effects.

## Methods

### Antibodies and Reagents

The anti-mouse CD3ε (145-2C11) and anti-mouse CD45.2 (104) antibodies were purchased from BD Biosciences. The anti-human CD3 (UCHT1), anti-mouse CD28 (3751), and anti-mouse IFN-γ (XMG1.2) antibodies were purchased from BioLegend. Recombinant human IL-2 was purchased from PeproTech, anti-human CD3ε (OKT3) was purchased from Novus Biologicals, anti-mouse Granzyme B (NGZB) and Brefeldin A was purchased from eBioscience, Annexin-V and 10x Annexin V Binding Buffer was purchased from BD Pharmingen, tetramethylrhodamine ethyl ester (TMRE) and puromycin dihydrochloride were purchased from Thermo Fisher, and SIINFEKL peptide (OVA) was ordered from BioPeptide. Anti-human CD28 (37407), mouse CXCL12 (460-SD/CF), mouse ICAM-1 (796-IC), and mouse CCL21 (457-6C/CF) were purchased from R&D. Anti-human Fc receptor binding inhibitor (polyclonal), anti-mouse CD16/32 Fc block (93), Protein A (10-1100), tetramethylrhodamine (TMRM), Cell Trace Violet, MitoTracker Green FM, and MitoTracker Red CMXRos were purchased from Invitrogen. Carbonyl cyanide 4-(trifluoromethoxy) phenylhydrazone (FCCP), all trans-Retinal, 2-Deoxy-D-glucose, and cobalt (II) chloride hexahydrate were purchased from Sigma. The immunoblot antibody anti-ß-actin (C4) HRP was purchased from Santa Cruz Biotechnology, anti-GFP (ab290) was purchased from Abcam, and peroxidase-conjugated AffiniPure Goat Anti-Rabbit IgG (111-035-144) was purchased from Jackson ImmunoResearch.

### Animals

Adult C57BL/6J and OT-I TCR transgenic (C57BL/6-Tg(TcraTcrb)1100Mjb/J) mice were purchased from the Jackson Laboratory and bred in our facility. Unless specified, it can be assumed that experiments were done with CD8^+^ T cells isolated from C57BL/6J mice. All mice were maintained in a pathogen-free environment in the University of Rochester animal facility and the animal experiments were approved by the University Committee on Animal Resources at the University of Rochester.

### Molecular Biology

The light-activated proton pump from *Leptosphaeria maculans* (Mac) fused to eGFP was amplified from the plasmid pFCK-Mac-GFP, a gift from Edward Boyden (Addgene plasmid #22223) (27)and fused to the N-terminal 187 amino acids of the *Immt* gene (mitofilin homologue) as previously described (23). The IMMT::Mac::GFP sequence was then cloned using restriction digestion into a pcDNA3.1 vector containing a CMV promoter (pBJB28 plasmid). Bionics cloned the Mitofilin::Mac::GFP sequence into a pMSCV vector, which was used to make retrovirus. The pcDNA3.1-GFP and pMigR1-GFP plasmids were used for negative control transfections in HEK293T cells and CD8^+^ T cells, respectively.

### Cell Culture

Isolated mouse CD8^+^ T cells were cultured in RPMI 1640 supplemented with 80 U/mL IL-2, 10% FBS, 100 U/mL penicillin (Gibco), 100 µg/mL streptomycin (Gibco), 2 mM L-glutamine (Gibco), 20 mM HEPES buffer (Gibco), 1% MEM Non-Essential Amino Acids (Gibco), and 50 µM ß-mercaptoethanol (Sigma-Aldrich). Isolated human T cells were cultured in TexMACS medium supplemented with 200 U/mL IL-2, 3% human AB serum, 100 U/mL penicillin, and 100 µg/mL streptomycin. HeLa cells, HEK293T cells, and EL-4 cells were cultured in DMEM supplemented with 10% FBS, 100 U/mL penicillin, 100 µg/mL streptomycin, 2 mM L-glutamine, 20 mM HEPES buffer, 1% MEM Non-Essential Amino Acids, and 50 µM β-mercaptoethanol. Geneticin (Gibco) was added to complete DMEM media for stable HEK293T cell lines at 1 mg/mL and for stable HeLa cell lines at 500 µg/mL. All cell types that expressed OptoMito-On were cultured in media supplemented with 7 µM all-*trans-*retinal (ATR) for at least 48 hours prior to any experiments. ATR is a cofactor that is required for photosensory transduction by the proton pump ‘Mac’ (24).

### T Cell Purification and Activation

Mouse CD8^+^ T cells were purified from single-cell suspensions of the spleen and lymph nodes of C57BL/6J or OT-I TCR transgenic mice. Single-cell suspensions were prepared by mechanical disruption in a cell strainer. CD8^+^ T cells were enriched by magnetic-bead depletion with rat anti-mouse MHC class II antibody (M5/114) and rat anti-mouse CD4 antibody (GK1.5), followed by sheep anti-rat IgG magnetic beads (Invitrogen 11035). Isolated CD8^+^ T cells were cultured up to 4 days in complete RPMI medium supplemented with 80 U/mL IL-2 following activation with plate-bound CD3ε Ab (6 µg/mL) and CD28 Ab (1.6 µg/mL) for 2 days. Human T cells were purified from healthy donor peripheral blood using the EasySep Direct Human T cell isolation kit (StemCell). Human T cell activation was achieved with plate-bound αCD3ε (1 µg/mL) and αCD28 (0.2 µg/mL) in complete TexMACS medium.

### Immunoblotting

The membrane was blocked with 5% nonfat milk in PBS for at least 1 hour at room temperature after proteins were transferred from precast 4-20% gels (BioRad). Blots were incubated for 1 hour at room temperature with 1:1000 of anti-GFP (ab290, Abcam) or 1:5000 β-actin HRP (SantaCruz). The membrane was then incubated with 1:5000 horseradish peroxidase-conjugated anti-rabbit IgG heavy and light chains (Jackson ImmunoResearch) for 1 hour at room temperature. Protein was detected using SuperSignal West Pico PLUS chemiluminescent reagent (Thermo).

### Metabolism Assays

The oxygen consumption rates (OCR) and extracellular acidification rates (ECAR) were measured in a Seahorse XFe 96 Analyzer with the Seahorse XF Cell MitoStress Test (Agilent). Measurements were made in L-15 medium supplemented with 2 mg/mL D-glucose under basal conditions and in response to 2 µM oligomycin, 2 µM FCCP, and 1 µM rotenone + 1 µM antimycin A. A BCA assay was used to normalize the Seahorse assay results in **Figure 1** and **Supplemental Figure 1** to total protein content. For all other Seahorse assays, the same number of cells was plated in each well. The Seahorse XFe96 Extracellular Flux Analyzer has been well characterized to determine the bioenergetics of T cells (54).

### ATP Assay

ATP measurements were performed with the CellTiter-Glo Luminescent Cell Viability Assay following the manufacturer’s instructions (Promega). The Lipofectamine 2000 DNA transfection protocol was used (Invitrogen). HEK293T cells expressing GFP were used as a negative control. Media was supplemented with 7 μM ATR. HEK293T cells were treated with 10 mM 2-DG 2 hours prior to light activation and 2-DG remained in the media during the 2-hour light activation. CD8^+^ T cells were sorted based on GFP expression. GFP negative cells were used as the mock negative control and the light activation was for 30 minutes. The light sources and conditions are described below (590 nm at 0.32 mW/mm^2^). The ATP assay in **Supplementary Figure 5B** was done with Day 6 OptoMito-On OT-I T cells enriched with 3 µg/mL puromycin. T cells were treated with 10 mM 2-DG for 30 minutes prior to a 30-minute light activation.

### Mitochondrial Staining

To label mitochondria, 100-500 nM MitoTracker Red CMXRos for HeLa cells was used. Images of live cells in L-15 on poly-L-lysine coated ΔTs were acquired on an inverted microscope. Images of fixed OptoMito-On expressing HeLa cells were taken on a confocal microscope. During CD8^+^ T cell activation, mitochondrial mass was determined with 200 nM MitoTracker Green FM and mitochondrial membrane potential with 20 nM TMRM. Background TMRM staining was subtracted by adding 10 μM FCCP while staining with TMRM. Cells were stained with MitoTracker Green FM or TMRM for 15 minutes at 37°C then stained with anti-mouse CD8 (Clone 53-6.7, BD Biosciences) or anti-human CD3 (UCHT1, Biolegend) followed by flow cytometry.

### Mitochondria Isolation

OptoMito-On HEK293T cell mitochondria were isolated from cells trypsinized from 5 T75 flasks using differential centrifugation in sucrose-based media. Collected cells were washed in PBS and pelleted in a 15 mL tube. The pelleted cells were resuspended in 2 mL cold mitochondria isolation buffer (10 mM HEPES, 1 mM EDTA, 320 mM sucrose, pH 7.2), transferred to a Dounce homogenizer, and homogenized with a pestle for 60 passes. The cell lysate was then transferred to a 2 mL Eppendorf tube and spun down at 700 g for 8 minutes. The supernatant contained the crude mitochondria fraction and was carefully removed then stored on ice. The pellet was resuspended in 500 μL isolation buffer, spun down at 700 g for 5 minutes, and the supernatant was pooled with the crude mitochondria fraction. The crude mitochondria fraction was then spun at 17,000 g for 11 minutes. The resulting supernatant contained the cytosolic fraction and the pellet contained mitochondria. The mitochondrial pellet was then resuspended in 50 μL of isolation buffer and protein concentration was quantified using the Folin-phenol method.

### Isolated Mitochondria TMRE Measurement

OptoMito-On isolated mitochondria (0.5 mg/mL) were stirred in mitochondrial respiration buffer (120 mM KCl, 25 mM sucrose, 5 mM MgCl_2_, 5 mM KH_2_PO_4_, 1 mM EGTA, 10 mM HEPES, 1 mg/mL FF-BSA, pH 7.35) at 37°C in the presence of 2 µM rotenone and 5 mM succinate where indicated. 20 nM tetramethylrhodamine ethyl ester (TMRE) was added to observe mitochondrial membrane potential in non-quench mode, as previously described (23). TMRE signal was measured by Cary Eclipse Fluorescence Spectrophotometer (Agilent Technologies) using a 335-620 nm excitation filter and a 550-1,100 nm emission filter. Illumination was performed continuously (0.02 mW/mm^2^, 540-600 nm GYX module, X-Cite LED1 by Excelitas, Waltham MA, USA) for 1 minute prior to fluorescence reading. After stable baseline measurements with or without succinate, 2 µM FCCP was added to completely depolarize mitochondrial proton motive force. Average fluorescence after FCCP addition was subtracted from the baseline to calculate the change in fluorescence (ΔF for conditions without succinate and ΔF_max_ for conditions with succinate). Data were then normalized to ΔF_max_ to show polarization of the Δψ_m_ by OptoMito-On relative to maximum endogenous polarization.

### *In* V*itro* T Cell Migration Imaging

Cell migration chambers (Millicell EZ slide eight-well glass, Millipore) were prepared by coating with Protein A (20 μg/mL), ICAM-1 (2.75 μg/mL), and CXCL12 (400 ng/mL) in PBS. Day 4 activated CD8^+^ T cells were plated in L-15 medium (Invitrogen, Leibovitz’s medium + 2 mg/mL D-glucose) in a 37°C chamber. Video microscopy was conducted using a TE2000-U microscope (Nikon) coupled to a CoolSNAP HQ CCD camera with a 10x objective and 0.45 numerical aperture. T cells were plated in L-15 medium at least 20 minutes at 37°C before imaging. Treatment of cells with inhibitors before imaging: CoCl_2_ treatment was overnight (~16 hours) and maintained in L-15 media; FCCP and oligomycin were added to the cells 20 minutes before the movie started when cells are plated. OptoMito-On and Mock or GFP T cell migration movies all received 500 nm illumination with a Texas Red filter. Bright field or DIC images were acquired every 15 seconds for 20 minutes.

### T Cell Migration Analysis

Migration analysis was performed in Volocity software (PerkinElmer). In order to select which T cell tracks to analyze in Volocity, we excluded cells that are smaller than 10 μm and greater than 200 μm. Additionally, static cells were ignored, broken tracks were automatically joined, and cell tracks less than 20 μm were excluded. We also omitted T cells that were migrating for less than 5 minutes of the 20-minute movie and removed cells that Volocity incorrectly tracked. The velocity (μm min^-1^), displacement (net displacement, μm), track length (total path length, μm), and meandering index (net displacement/track length) of each cell track was measured (55). The percentage of migrating cells was calculated as the number of cells migrating 5-20 µm/min divided by the total number of cells in the field of view during a 20-minute movie.

### OptoMito-On Expression in T Cells

Retroviruses were generated using the Phoenix-ecotropic packaging cell line (ATCC). For retroviral transductions, Phoenix cells were transfected to produce retrovirus using the calcium phosphate transfection method. Collected supernatant was concentrated with Retro-X Concentrator (Takara). Isolated mouse CD8^+^ T cells were transduced one day after isolation in the presence of 8 μg/mL polybrene. Cells were then sorted on Day 3 or 4 based on GFP signal and used for experiments on Day 4-6. Typical transduction efficiency for OptoMito-On is 30%. Considerable time was spent optimizing the retrovirus transduction efficiency and the best efficiency was 45-50%. OptoMito-On or GFP transduced CD8^+^ T cells were not sorted for the granzyme B assay; instead GFP+ cells were gated on during flow cytometry analysis. At least 30% of CD8^+^ T cells were OptoMito-On+. For the ATP assay **(Supplementary Figure 5A**), OptoMito-On T cells were enriched with 3 μg/mL puromycin, resulting in at least 90% OptoMito-On+ T cells. All media for experiments using OptoMito-On expressing T cells or HEK293T cells was supplemented with 7 μM ATR at least two days before cells were used for experiments.

### Antibody Array

Full Moon BioSystems Cytoskeleton Phospho Antibody Array (PCP141) was used following the manufacturer’s instructions. OptoMito-On expressing CD8^+^ T cells were sorted based on GFP expression and 150,000 cells were plated on ICAM-1 + CXCL12 coated 8-well dishes. T cells either received no light activation or received the same light activation as was done for *in vitro* T cell imaging (Texas Red filter at 630 nm). Immediately after the 20-minute movie +/- light activation, L-15 was aspirated to remove any unattached cells, followed by the addition of RIPA buffer, with phosphate & protease inhibitors added, to lyse the attached migrating cells. Cell lysate was pooled from multiple movies in order to have enough protein to complete three replicates of the antibody array and quantified with a BCA assay. The antibody array results were quantified using ImageJ, and the average fold change in protein expression from dark to light conditions was calculated to make a heat map.

### Co-Culture Assay

Activated OT-I CD8^+^ T cells transduced with OptoMito-On or GFP retrovirus were sorted on Day 3 or Day 4 of activation. 1 - 2 hours after cell sorting, 100,000 T cells were plated in each well of glass bottom 96-well plates in complete RPMI media. One plate remained in the dark, while the second plate was illuminated with 530 nm light at 0.0075 mW/mm^2^ for 16-17 hours. EL-4 cells were pulsed with 1 μg/mL SIINFEKL (OVA) peptide and stained with 5 μM Cell Trace Violet for 20 minutes at 37°C, then washed in PBS before adding 100,000 EL-4 cells to the already plated CD8^+^ T cells in complete RPMI media. The co-culture was done at a 1:1 ratio for 3 hours in duplicate for each experiment. The illuminated plate was only briefly removed from the LED array when EL-4 cells were added. At the completion of the co-culture, cells were collected, washed in cold PBS, then stained with Annexin-V APC in 1x Annexin V binding buffer for flow cytometry analysis. Cell Trace Violet positive cells were gated on to specifically look at the Annexin-V staining of EL-4 cells.

### Glucose Consumption Assay

Activated OT-I CD8^+^ T cells transduced with OptoMito-On or GFP retrovirus were sorted on Day 3 or Day 4 of activation. A couple hours after cell sorting, T cells were plated in each well of glass bottom 96-well plates in 100 μL Leibovitz’s media, without any added glucose. One plate remained in the dark, while the second plate was illuminated with 530 nm light at about 0.013 mW/mm^2^ for 1 hour. 1 mM 2-DG was added to each well before the 1-hour incubation in order to measure the glucose consumption rate with the Glucose Uptake-Glo Assay (Promega). After the 1-hour incubation, 50 μL of stop buffer was added to each well, then 75 μL of the cells were pipetted into a white 96-well plate. The remainder of the assay strictly followed the manufacturer’s instructions. In order to subtract background signal, 2-DG wasn’t added to control wells with the same number of cells.

### Light Sources

A 540-600 nm GYX module, X-Cite LED1 by Excelitas, Waltham MA, USA was used at 0.02 mW/mm^2^ to illuminate isolated mitochondria for the TMRE traces. An Amuza 590 nm LED array was used to illuminate HEK293T cells and CD8^+^ T cells with light for the ATP assay. Each well of a glass-bottom 96-well plate (MatTek, P96G-1.5-5-F) received 590 nm light at 0.32 mW/mm^2^ at 1 hertz, with a 500-ms light/500-ms dark cycle. An Amuza 530 nm LED array was used to illuminate OT-I CD8^+^ T cells for the overnight Granzyme B experiment (0.0045 mW/mm^2^), co-culture assay (0.0075 mW/mm^2^), and glucose consumption assay (0.013 mW/mm^2^). Each well of a glass-bottom 96-well plate received 530 nm light at 1 hertz with a 500-ms light/500-ms dark cycle. Light activation for *in vitro* T cell migration assays were performed on a TE2000-U microscope (Nikon, described above) using the Texas Red filter (630 nm) at approximately 0.75 mW/mm^2^ with a 5-s light/5-s dark cycle using a Sola light engine (Nikon). The light intensities were determined with an energy meter console connected to a photodiode power sensor (PM100D, S130VC, ThorLabs).

### Statistical Analyses

Assuming normal distribution, for comparison of non-paired experimental groups an unpaired t-test was utilized. Comparisons for more than two groups employed a One-way ANOVA or a Two-way ANOVA for more than 1 level of comparison. ANOVA tests were accompanied with Bonferroni’s post-hoc test. Additionally, Pearson’s correlation and a paired t-test was used when appropriate. Differences with a p-value < 0.05 were considered statistically significant. All statistical tests were performed with GraphPad Prism (v9). Means ± standard error of the mean (SEM) are shown.

### Study Approval

All mouse procedures were approved by the University of Rochester Committee on Animal Resources under protocol UCAR-2008-096E and followed Institutional Animal Care and Use Committee guidelines. The Human Research Studies Review Board of the University of Rochester approved this study.

## Data Availability Statement

The raw data supporting the conclusions of this article will be made available by the authors, without undue reservation.

## Ethics Statement

The animal study was reviewed and approved by University Committee on Animal Resources at the University of Rochester.

## Author Contributions

AA conducted the majority of the experiments with the help of BB who completed the cloning of the OptoMito-On construct into the pcDNA3.1 vector, isolated mitochondria, and collected TMRE traces. KL performed the antibody arrays. RW helped with human T cell assays. MK and AW conceived the idea of OptoMito-On and directed this study. K-DK helped design and optimize the light activation experiments and co-culture assay. AA and MK wrote the manuscript with suggestions from all the authors. All authors contributed to the article and approved the submitted version.

## Funding

This project was financially supported through grants from the National Institute of Health (T32AI007285 (AA), P01AI102851 (MK), R01AI147362 (MK & RW), R01NS115906 (AW), and R21CA242843 (MK & AW). This project was financially supported by the National Research Council of Science and Technology (NST) grant through the Korean government (MSIP) (CRC-16-01-KRICT).

## Conflict of Interest

The authors declare that the research was conducted in the absence of any commercial or financial relationships that could be construed as a potential conflict of interest.

## Supplementary Material

Supplementary Figure 1 Overall CD8^+^ T cell metabolism increases after activation. The complete ECAR trace **(A)**, the basal OCR **(B)**, the maximum OCR **(C)**, and the spare respiratory capacity **(D)** of naïve and activated CD8^+^ T cells, measured with the Seahorse MitoStress Test and normalized to protein content with a BCA assay. **(A)**: data shown as mean ± SEM, error bars fall within symbols, analyzed by 2way ANOVA with a Bonferroni post-test; **(B–D)**: data shown as mean ± SEM and analyzed by Welch’s t-test. **(E)** Increase in the percent of Annexin-V+ activated mouse CD8^+^ T cells after treatment with 1 µM Rotenone + Antimycin A, 2 µM FCCP, or 2 µM Oligomycin for 1, 3, or 6 hours (n = 3).

Supplementary Figure 2 Migrating CD8^+^ T cells have increased glycolysis. The complete ECAR trace **(A)**, the basal ECAR **(B)**, the maximum OCR **(C)**, and the spare respiratory capacity **(D)** of activated CD8^+^ T cells, measured with the Seahorse MitoStress Test. **(A)**: data shown as mean ± SEM, error bars fall within symbols; **(B–D)**: data shown as mean ± SEM and analyzed by One-Way ANOVA with a Bonferroni post-test.

Supplementary Figure 3 CD8^+^ T cell migration capacity is decreased in hypoxic conditions. **(A)** The complete OCR trace and **(B)** the basal OCR measured with the Seahorse MitoStress Test, of activated CD8^+^ T cells treated with the hypoxia inducer, cobalt chloride hexahydrate (CoCl_2_) overnight (n = 6 wells per group, error bars fall within symbols). **(C)** The complete ECAR trace and **(D)** the basal ECAR measured with the Seahorse MitoStress Test. **(E)** The overall track velocity of activated CD8^+^ T cells treated with or without CoCl_2_ and migrating on ICAM-1 + CXCL12 (includes all tracked cells migrating less than 15 µm/min). **(F)** The velocity of actively migrating CD8^+^ T cells. **(G)** The percentage of migrating CD8^+^ T cells (the number of cells migrating 5-20 µm/min divided by the total number of cells in the field of view during a 20-minute movie, n = 2 movies). **(H)** Flow cytometry results of activated CD8^+^ T cells treated with CoCl_2_ overnight and stained for Annexin-V. **(A–G)**: data shown as mean ± SEM and analyzed by One-Way ANOVA with a Bonferroni post-test.

Supplementary Figure 4 OptoMito-On is expressed in mitochondria. CD8^+^ T cells, HEK293T cells, and HeLa cells expressing OptoMito-On were stained with MitoTracker Red and images were taken on an inverted microscope. Then the Pearson’s correlation coefficient was calculated in ImageJ software for the entire cell body of each cell. Data shown as mean ± SEM.

Supplementary Figure 5 OptoMito-On does not impact glycolysis. **(A)** ATP assay with OptoMito-On OT-I T cells treated with or without 10 mM 2-DG. Same light activation setup as **Figure 4C**. Data shown as mean ± SEM (n = 3-4). **(B)** Sorted OT-I T cells expressing either OptoMito-On or GFP were plated in Leibovitz’s media, illuminated with 530 nm for 1 hour or kept in the dark, and then the Glucose Uptake-Glo Assay protocol was followed. Data shown as mean ± SEM and analyzed by One-Way ANOVA with a Bonferroni post-test (representative of three experiments; RLU: relative luminescence units).

Movie 1 Representative movie of CD8^+^ T cells expressing GFP migrating on ICAM-1 + CXCL12 with 500 nm illumination.

Movie 2 Representative movie of CD8^+^ T cells expressing OptoMito-On migrating on ICAM-1 + CXCL12 with 500 nm illumination.
