# Supplementary material for: Optical Control of CD8+ T Cell Metabolism and Effector Functions
Source: Front Immunol. 2021 Jun 3;12:666231. doi: 10.3389/fimmu.2021.666231 (PMC8209468; doi:10.3389/fimmu.2021.666231)
Supplement: Supplementary Figure 1 — Overall CD8+ T cell metabolism increases after activation. The complete ECAR trace (A), the basal OCR (B), the maximum OCR (C), and the spare respiratory capacity (D) of naïve and activated CD8+ T cells, measured with the Seahorse MitoStress Test and normalized to protein content with a BCA assay. (A): data shown as mean ± SEM, error bars fall within symbols, analyzed by 2way ANOVA with a Bonferroni post-test; (B–D): data shown as mean ± SEM and analyzed by Welch’s t-test. (E) Increase in the percent of Annexin-V+ activated mouse CD8+ T cells after treatment with 1 µM Rotenone + Antimycin A, 2 µM FCCP, or 2 µM Oligomycin for 1, 3, or 6 hours (n = 3). [file Image_1.pdf]

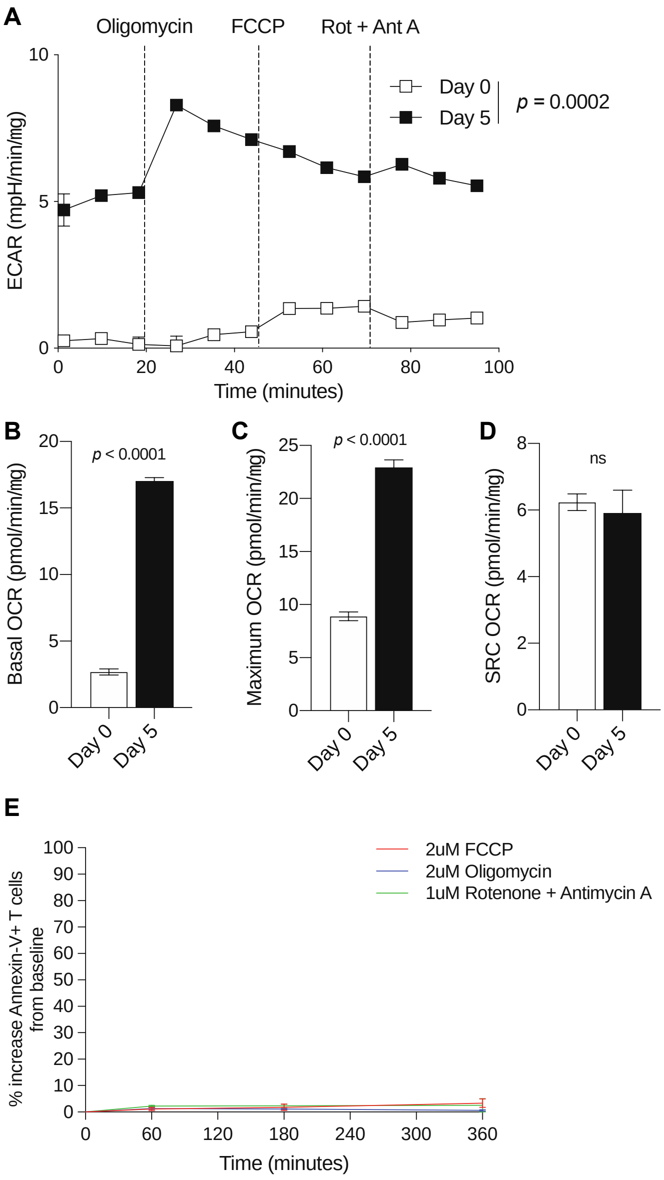

Supplemental Figure 1. Overall CD8$^+$ T cell metabolism increases after activation. The complete ECAR trace (**A**), the basal OCR (**B**), the maximum OCR (**C**), and the spare respiratory capacity (**D**) of naïve and activated CD8$^+$ T cells, measured with the Seahorse MitoStress Test and normalized to protein content with a BCA assay. A: data shown as mean ± SEM, error bars fall within symbols, analyzed by 2way ANOVA with a Bonferroni post-test; B-D: data shown as mean ± SEM and analyzed by Welch's t-test. (**E**) Increase in the percent of Annexin-V+ activated mouse CD8$^+$ T cells after treatment with 1 µM Rotenone + Antimycin A, 2 µM FCCP, or 2 µM Oligomycin for 1, 3, or 6 hours (n = 3). (ns: not significant)
