# Supplementary material for: Optical Control of CD8+ T Cell Metabolism and Effector Functions
Source: Front Immunol. 2021 Jun 3;12:666231. doi: 10.3389/fimmu.2021.666231 (PMC8209468; doi:10.3389/fimmu.2021.666231)
Supplement: Supplementary Figure 2 — Migrating CD8+ T cells have increased glycolysis. The complete ECAR trace (A), the basal ECAR (B), the maximum OCR (C), and the spare respiratory capacity (D) of activated CD8+ T cells, measured with the Seahorse MitoStress Test. (A): data shown as mean ± SEM, error bars fall within symbols; (B–D): data shown as mean ± SEM and analyzed by One-Way ANOVA with a Bonferroni post-test. [file Image_2.pdf]

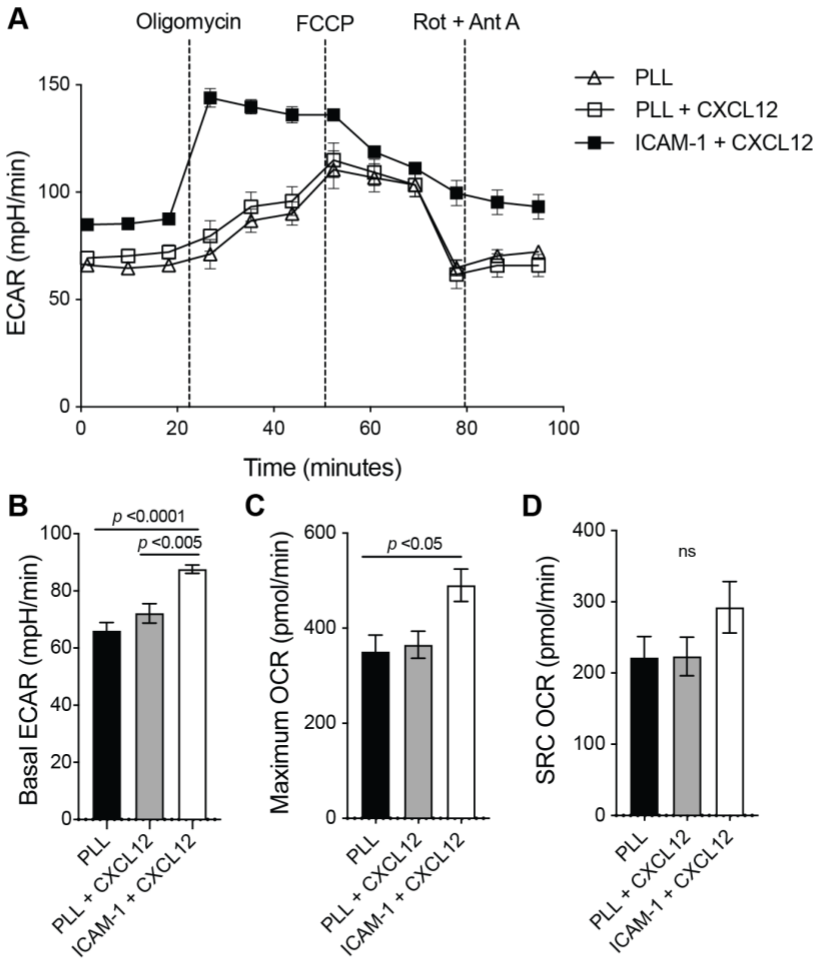

Supplemental Figure 2. Migrating CD8$^+$ T cells have increased glycolysis. The complete ECAR trace (**A**), the basal ECAR (**B**), the maximum OCR (**C**), and the spare respiratory capacity (**D**) of activated CD8$^+$ T cells, measured with the Seahorse MitoStress Test. A: data shown as mean ± SEM, error bars fall within symbols; B-D: data shown as mean ± SEM and analyzed by One-Way ANOVA with a Bonferroni post-test. (ns: not significant)
