# Supplementary material for: Optical Control of CD8+ T Cell Metabolism and Effector Functions
Source: Front Immunol. 2021 Jun 3;12:666231. doi: 10.3389/fimmu.2021.666231 (PMC8209468; doi:10.3389/fimmu.2021.666231)
Supplement: Supplementary Figure 3 — CD8+ T cell migration capacity is decreased in hypoxic conditions. (A) The complete OCR trace and (B) the basal OCR measured with the Seahorse MitoStress Test, of activated CD8+ T cells treated with the hypoxia inducer, cobalt chloride hexahydrate (CoCl2) overnight (n = 6 wells per group, error bars fall within symbols). (C) The complete ECAR trace and (D) the basal ECAR measured with the Seahorse MitoStress Test. (E) The overall track velocity of activated CD8+ T cells treated with or without CoCl2 and migrating on ICAM-1 + CXCL12 (includes all tracked cells migrating less than 15 µm/min). (F) The velocity of actively migrating CD8+ T cells. (G) The percentage of migrating CD8+ T cells (the number of cells migrating 5-20 µm/min divided by the total number of cells in the field of view during a 20-minute movie, n = 2 movies). (H) Flow cytometry results of activated CD8+ T cells treated with CoCl2 overnight and stained for Annexin-V. (A–G): data shown as mean ± SEM and analyzed by One-Way ANOVA with a Bonferroni post-test. [file Image_3.pdf]

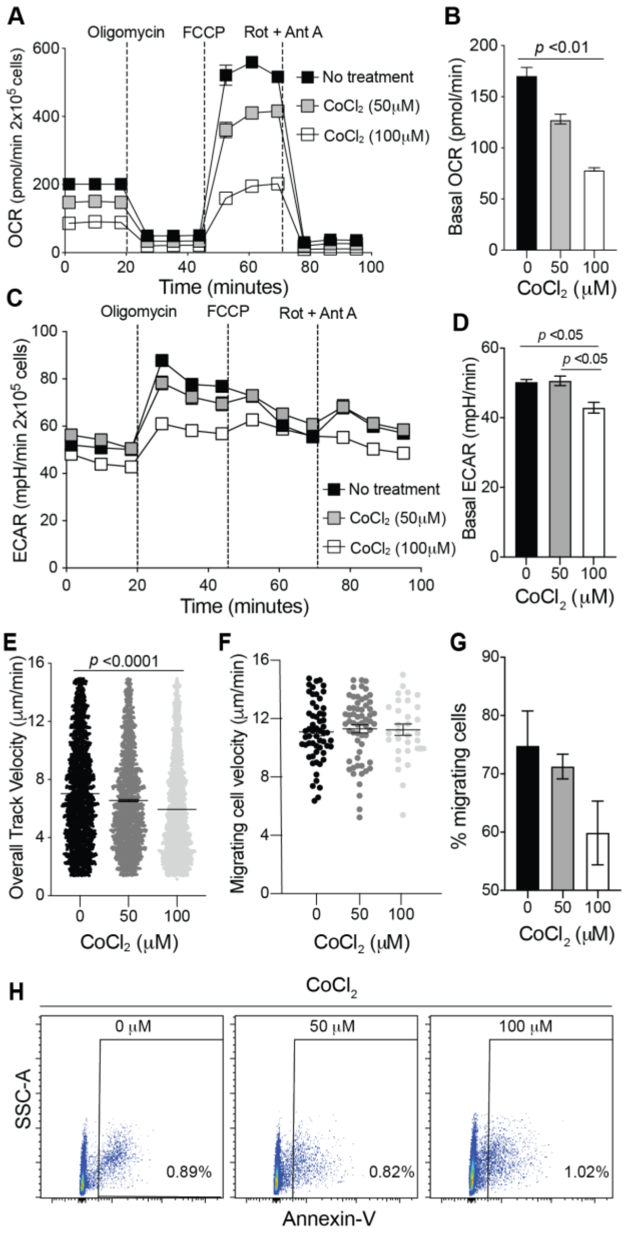

Supplemental Figure 3. CD8$^+$ T cell migration capacity is decreased in hypoxic conditions. (**A**) The complete OCR trace and (**B**) the basal OCR measured with the Seahorse MitoStress Test, of activated CD8$^+$ T cells treated with the hypoxia inducer, cobalt chloride hexahydrate ($CoCl_2$) overnight (n = 6 wells per group, error bars fall within symbols). (**C**) The complete ECAR trace and (**D**) the basal ECAR measured with the Seahorse MitoStress Test. (**E**) The overall track velocity of activated CD8$^+$ T cells treated with or without $CoCl_2$ and migrating on ICAM-1 + CXCL12 (includes all tracked cells migrating less than 15 μm/min). (**F**) The velocity of actively migrating CD8$^+$ T cells. (**G**) The percentage of migrating CD8$^+$ T cells (the number of cells migrating 5-20 μm/min divided by the total number of cells in the field of view during a 20-minute movie, n = 2 movies). (**H**) Flow cytometry results of activated CD8$^+$ T cells treated with $CoCl_2$ overnight and stained for Annexin-V. A-G: data shown as mean ± SEM and analyzed by One-Way ANOVA with a Bonferroni post-test.
