# Supplementary material for: Optical Control of CD8+ T Cell Metabolism and Effector Functions
Source: Front Immunol. 2021 Jun 3;12:666231. doi: 10.3389/fimmu.2021.666231 (PMC8209468; doi:10.3389/fimmu.2021.666231)
Supplement: Supplementary Figure 4 — OptoMito-On is expressed in mitochondria. CD8+ T cells, HEK293T cells, and HeLa cells expressing OptoMito-On were stained with MitoTracker Red and images were taken on an inverted microscope. Then the Pearson’s correlation coefficient was calculated in ImageJ software for the entire cell body of each cell. Data shown as mean ± SEM. [file Image_4.pdf]

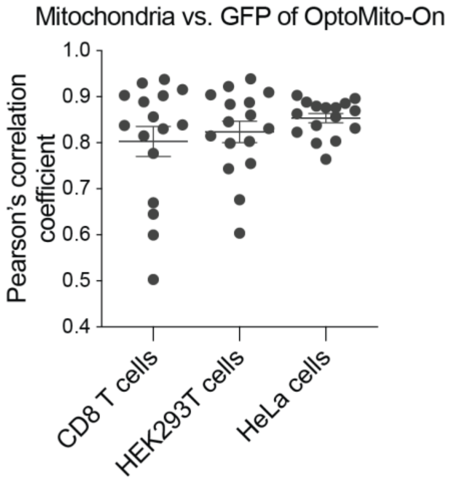

Supplemental Figure 4. OptoMito-On is expressed in mitochondria. $CD8^+$ T cells, HEK293T cells, and HeLa cells expressing OptoMito-On were stained with MitoTracker Red and images were taken on an inverted microscope. Then the Pearson's correlation coefficient was calculated in ImageJ software for the entire cell body of each cell. Data shown as mean ± SEM.
