# Supplementary material for: Optical Control of CD8+ T Cell Metabolism and Effector Functions
Source: Front Immunol. 2021 Jun 3;12:666231. doi: 10.3389/fimmu.2021.666231 (PMC8209468; doi:10.3389/fimmu.2021.666231)
Supplement: Supplementary Figure 5 — OptoMito-On does not impact glycolysis. (A) ATP assay with OptoMito-On OT-I T cells treated with or without 10 mM 2-DG. Same light activation setup as Figure 4C . Data shown as mean ± SEM (n = 3-4). (B) Sorted OT-I T cells expressing either OptoMito-On or GFP were plated in Leibovitz’s media, illuminated with 530 nm for 1 hour or kept in the dark, and then the Glucose Uptake-Glo Assay protocol was followed. Data shown as mean ± SEM and analyzed by One-Way ANOVA with a Bonferroni post-test (representative of three experiments; RLU: relative luminescence units). [file Image_5.pdf]

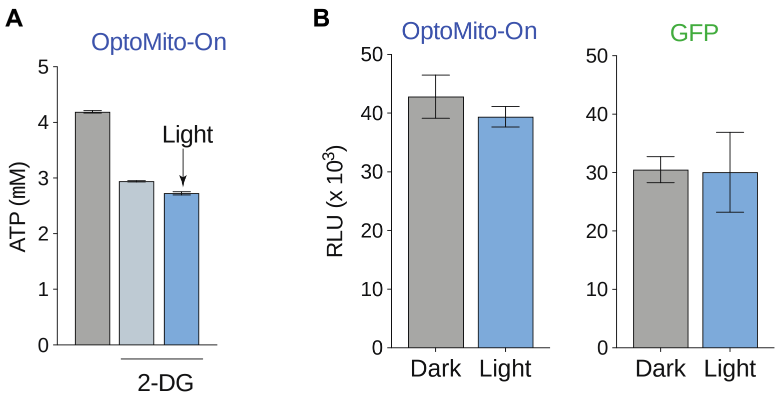

Supplemental Figure 5. OptoMito-On does not impact glycolysis. (**A**) ATP assay with OptoMito-On OT-I T cells treated with or without 10 mM 2-DG. Same light activation setup as Figure 4C. Data shown as mean ± SEM (n = 3-4). (B) Sorted OT-I T cells expressing either OptoMito-On or GFP were plated in Leibovitz's media, illuminated with 530 nm for 1 hour or kept in the dark, and then the Glucose Uptake-Glo Assay protocol was followed. Data shown as mean ± SEM and analyzed by One-Way ANOVA with a Bonferroni post-test (representative of three experiments; RLU: relative luminescence units).
